# Robust Stereo Visual-Inertial Odometry Using Nonlinear Optimization

**DOI:** 10.3390/s19173747

**Published:** 2019-08-29

**Authors:** Shujun Ma, Xinhui Bai, Yinglei Wang, Rui Fang

**Affiliations:** School of Mechanical Engineering and Automation, Northeastern University, Shenyang 110819, China

**Keywords:** localization, visual-inertial SLAM, state estimation, simultaneous localization and mapping

## Abstract

The fusion of visual and inertial odometry has matured greatly due to the complementarity of the two sensors. However, the use of high-quality sensors and powerful processors in some applications is difficult due to size and cost limitations, and there are also many challenges in terms of robustness of the algorithm and computational efficiency. In this work, we present VIO-Stereo, a stereo visual-inertial odometry (VIO), which jointly combines the measurements of the stereo cameras and an inexpensive inertial measurement unit (IMU). We use nonlinear optimization to integrate visual measurements with IMU readings in VIO tightly. To decrease the cost of computation, we use the FAST feature detector to improve its efficiency and track features by the KLT sparse optical flow algorithm. We also incorporate accelerometer bias into the measurement model and optimize it together with other variables. Additionally, we perform circular matching between the previous and current stereo image pairs in order to remove outliers in the stereo matching and feature tracking steps, thus reducing the mismatch of feature points and improving the robustness and accuracy of the system. Finally, this work contributes to the experimental comparison of monocular visual-inertial odometry and stereo visual-inertial odometry by evaluating our method using the public EuRoC dataset. Experimental results demonstrate that our method exhibits competitive performance with the most advanced techniques.

## 1. Introduction

In recent years, with the advancement of sparse nonlinear optimization theory, camera technology, and computing performance, Visual Simultaneous Localization And Mapping [VSLAM] technology has achieved tremendous development [1,2]. Visual SLAM approaches have been widely used and researched because of their simple equipment and remarkable effects. Real-time and robust state estimation plays a significant role in robotics. Accurate state estimation is of crucial importance in a variety of intelligent applications, such as robot autonomy, Augmented Reality [AR] and Virtual Reality (VR). 

The vision-based state estimation method is able to estimate the 6-Degrees-Of-Freedom (6-DOF) state of sensors simultaneously and reconstruct a three-dimensional (3D) map of the surrounding environment. Many works based on nonlinear optimization have been reported, including SVO [3], LSD-SLAM [4], DSO [5], ORB-SLAM [6,7]. The ORB-SLAM system supports monocular cameras, stereo cameras, and depth cameras. The system is also designed based on the idea of PTAM [8], which is divided into three threads: tracking, mapping and loop closing. However, due to the existence of observation noise, the pose estimation of the feature points in space contains uncertainty, leading to localization errors in Visual Odometry. The position change between the current frame and the previous one can be calculated and continuously accumulated by Visual Odometry in order to realize the estimation of the motion process of a robot. During this process, the error of each estimation is collected, thus limiting the accuracy of Visual Odometry over time.

An Inertial Measurement Unit (IMU) is composed of an accelerometer and a gyroscope. The pose of the robot can be calculated and the three-dimensional heading information can be estimated, respectively, by the accelerometer and gyroscope in the IMU. However, the navigation errors caused by the low-frequency noise of the IMU work process will accumulate over time due to the integral operation, and the accuracy will easily drift, resulting in inaccurate pose estimation. A general and effective solution to these problems is to fuse visual and IMU measurements using a filter-based or optimization-based system. During the fusing process, IMU and Vision are combined to form a Visual-Inertial Odometry, which not only takes advantage of the flexibility of the visual method and is adaptable to a wide range of scenes, but also utilizes the high-precision features of the IMU in the short term. Therefore, research into the SLAM algorithm based on visual and inertial sensors is of great significance and application value, allowing robots to perceive the surrounding environment in order to obtain localization information.

In Visual-Inertial Odometry (VIO), the easiest way to handle visual and inertial measurements is to loosely couple the sensor fusion [9], whereby the IMU is considered to be a separate module for assisting in visual pose estimation, and then is fused by an extended Kalman filter (EKF). In comparison, tightly coupled visual-inertial algorithms can mainly be classified into two types: EKF-based algorithms [10,11,12] and optimization-based algorithms [13,14,15]. For example, MSCKF [16] is a popular EKF-based VIO method. MSCKF keeps several previous camera poses in the state vector, and arranges them in chronological order, which is also known as a sliding window. If a feature point is observed in several poses of the sliding window, the constraint will be established between these poses, and KF updates will be performed. A disadvantage of using a filter-based approach is that it can lead to suboptimal results due to the state of early linearization estimates.

Meanwhile, with the deepening of research and the improvement of computer performance, optimization-based methods have commonly been used in visual-inertial SLAM systems to ensure higher accuracy. A full smoothing method [17] for estimating the entire state history is described by solving a substantial nonlinear optimization problem. Although the outlook is promising, its computational complexity is high, and its real-time performance will gradually decrease as the map grows. Recently, the work proposed in [14] applied a keyframe-based approach to fuse visual-inertial measurements. The use of a sliding window and marginal technology [14,15] ensures the real-time operation of the system, and it has achieved remarkable success. Additionally, the IMU pre-integration technique proposed in [18] can avoid the repeated calculation of the integral when the linearization point changes. Mur-Artal et al. [15] suggested a VI-SLAM system based on their original ORB-SLAM, which originated from the pre-integration ideas of Forster [18]. Qin et al. [19] demonstrated a new Visual-Inertial System (VINS) which is also a complete VI-SLAM system based on tight coupling and nonlinear optimization. The initialization process is robust to unknown states and has excellent application potentials in the field of UAV navigation control.

Very few VIO solutions are planned for stereo or multi-camera systems [14,20,21] compared to the large amount of work that has been carried out for monocular systems. This could partly be due to the costs associated with processing additional images and matching features. Leutenegger et al. [14] proposed a complete optimization framework for multi-camera VIO that can run in real-time. Usenko et al. [20] introduced direct methods into stereo VIO to further improve accuracy. The tested dataset in [20] was acquired using a stereo visual inertial camera that was processed offline. IMU pre-integration was further improved in the stereo VIO in [20]; however, this solution is not suitable for practical applications. Liu et al. [22] proposed a stereo VIO assembled with three separate Kalman filters, including an attitude filter, an orientation filter, and a position filter. Wang et al. [23] proposed a stereo direct sparse odometry, which maintains high accuracy while achieving real-time pose estimation. Xiong et al. [24] presented a dual-stage EKF-based algorithm for the robust stereo VIO. Zheng et al. [25] introduced a tightly coupled filtering-based stereo VIO system using both points and lines. Some VIO algorithms have been explored in other areas, such as initialization [26], online calibration [27], optimization [28,29], and camera type [30]. These solutions are either based on optimization methods or filtering methods. For the optimization-based methods, they require a powerful CPU for real-time use and cannot run on low-cost devices such as embedded devices. For the filtering-based methods, the localization accuracy may not be enough. 

In this paper, we summarize our contributions as follows:

- The FAST feature detector was employed for its efficiency, and features were tracked by the KLT sparse optical flow algorithm to decrease the cost of computation.
- Stereo matching was performed, which can remove outliers in the stereo matching and feature tracking steps leading to reduction of the mismatch of feature points and improvement of the robustness and accuracy of the system.
- A tightly coupled nonlinear optimization method was employed to combine pre-integrated IMU measurements with visual measurements of stereo cameras, and thus a highly accurate and robust visual-inertial odometry was achieved, which can run in real-time on devices such as drones.
- The proposed method was extensively evaluated and validated in comparison to state-of-the-art open source VIO methods (including OKVIS [14], VINS-MONO [19] and S-MSCKF [16]) by using the EuRoC dataset.

## 2. Visual-Inertial Odometry Overview

**Figure 1:**
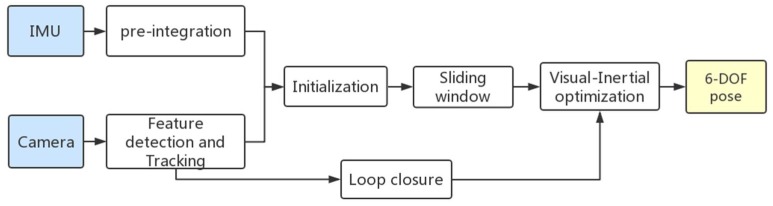
A block diagram illustrating our Visual-Inertial optimization framework.

The schematic depiction of the Visual-Inertial optimization framework is shown in Figure 1. The input of the system is the image acquired by the stereo cameras and the acceleration and angular velocity measured by the IMU. The output is a 6-degree-of-freedom pose with real scale, i.e., the trajectory of the inertial camera (robot) motion. The system starts with Visual-Inertial Preliminaries (Section 3), where features are extracted and tracked, and pre-integrate IMU measurements between two consecutive frames. Then the initialization preprocessing is introduced (Section 4). The inertial pose and visual pose are combined by visual inertia joint initialization to obtain the initial estimated value of the system, including pose, velocity, gyroscope bias, gravity vector. These values will iteratively and sequentially be updated by the tightly coupled visual-inertial odometry (Section 5). Finally, the 6-DOF pose can be obtained.

In this work, we consider ${\left(\cdot \right)}_{C}$ to be the camera frame, ${\left(\cdot \right)}_{B}$ to be the body frame, and we regard the IMU frame as the body frame. The matrix R represents rotation. $R_{WB}$ represents the rotation from the body frame to the world frame. $P_{WB}$ is translation from the body frame to the world frame.

## 3. Visual-Inertial Preliminaries

This section gives the preliminary steps for stereo visual measurements and the inertial model. For visual measurements, features are tracked in real-time using the KLT optical flow algorithm. For IMU measurements, two consecutive frames are pre-integrated. 

### 3.1. Visual Processing Frontend

In the Visual-Inertial odometry, the camera needs to track the pixel points of the captured image frame, and the pixel points need to be the feature points extracted from the previous and subsequent frames. However, there is only a small part of the overlap between successive image frames in many cases, and it would be inappropriate to match all the pixels, due to the massive demand for computing resources. Additionally, because the feature points are matched through the calculation process of the camera pose, it is unnecessary to extract many feature points. Therefore, in visual odometry, only the feature points from part of the image—i.e., the points with the most noticeable features in the image (corner points, points with sharp changes in brightness or at the edge of the contour)—are extracted and subsequently tracked.

In this paper, the feature points are extracted by the FAST [31] feature detector, and the feature tracking method is the KLT [32] optical flow algorithm. Through experiments, it is found that the KLT optical flow method takes up less CPU resources than the descriptor-based method, making the optical flow method more advantageous in robot applications. The KLT optical flow method was also used for stereo feature matching to save computing resources. Optical flow tracking is able to obtain the feature point coordinates of cam1 (the right camera of the stereo), which correspond to stereo cam0. Then, based on the principle of epipolar geometry, the following constraint can be defined as

(1) $$X_{2}^{T}EX_{1}=0$$

where $X_{1}$ and $X_{2}$ are the normalized plane coordinates of the feature points, $E=t^{\wedge }R$ is the essential matrix, and $t^{\wedge }$ is the antisymmetric matrix corresponding to the t vector. t and R are the translation vector and rotation matrix between the stereo cameras, respectively, which can be obtained from the initial calibration.

During the measurement, $X_{2}^{T}EX_{1}$ are not strictly equal to zero, due to the presence of noise. There is an error in this process, and if the error is greater than a certain threshold, it is marked as an outlier. $X_{1},X_{2}$ are obtained by LK optical flow tracking according to the original image, and the accuracy of optical flow tracking is verified by the constraint (relative poses *R* and *t*) between stereo cameras. Therefore, the effect of removing outliers can be achieved. Meanwhile, several grids were added to the image, which was assigned with feature points. The number of feature points attached to each grid was checked to ensure that it did not exceed the maximum value of grid feature points.

In the visual processing frontend, outlier rejection was performed using two-point RANSAC similar to (16) in temporal tracking. Assuming that the normalized coordinates of the corresponding points of the previous and current frames are X(x,y,1), $X_{2}\left(x_{2},y_{2},1\right)$, the following constraints can be established based on epipolar geometry:

(2) $$X_{2}^{T}t^{\wedge }RX=0$$

where *R* is obtained from the average angular velocity of the IMU. When $X_{1}=RX$, the coordinate system is unified into one coordinate system.

(3) $$\left[x_{2}\;\;\;y_{2}\;\;\;1\right]\cdot \left[\begin{array}{ccc} 0 & -t_{z} & t_{y}\\ t_{z} & 0 & -t_{x}\\ -t_{y} & t_{x} & 0 \end{array}\right]\cdot \left[\begin{array}{c} x_{1}\\ y_{1}\\ 1 \end{array}\right]\;=\;0$$

Equation (3) can be expanded as

(4) $$\left[y_{1}-y_{2}\;\;-\left(x_{1}-x_{2}\right)\;\;x_{1}y_{2}-x_{2}y_{2}\right]\cdot \left[\begin{array}{c} t_{x}\\ t_{y}\\ t_{z} \end{array}\right]\;=\;0$$

According to the RANSAC principle, two matching points of the previous frame and the current frame are arbitrarily selected, and the outliers with large errors are removed.

(5) $$\left[\begin{array}{ccc} y_{1}-y_{2} & -\left(x_{1}-x_{2}\right) & x_{1}y_{2}-x_{2}y_{2}\\ y_{3}-y_{4} & -\left(x_{3}-x_{4}\right) & x_{3}y_{4}-x_{4}y_{3} \end{array}\right]\cdot \left[\begin{array}{c} t_{x}\\ t_{y}\\ t_{z} \end{array}\right]\;=\;{\left[\begin{array}{c} A_{x}\\ A_{y}\\ A_{z} \end{array}\right]}^{T}\cdot \left[\begin{array}{c} t_{x}\\ t_{y}\\ t_{z} \end{array}\right]\approx \left[\begin{array}{c} 0\\ 0 \end{array}\right]$$

where $A_{x}=\left[y_{1}-y_{2}\;y_{3}-y_{4}\right]$. The following three formulas are available:

(6) $${\left[\begin{array}{c} A_{x}\\ A_{y} \end{array}\right]}^{T}\cdot \left[\begin{array}{c} t_{x}\\ t_{y} \end{array}\right]\approx \;A_{z}\cdot t_{z}\;{\left[\begin{array}{c} A_{x}\\ A_{z} \end{array}\right]}^{T}\cdot \left[\begin{array}{c} t_{x}\\ t_{z} \end{array}\right]\approx \;A_{y}\cdot t_{y}\;{\left[\begin{array}{c} A_{y}\\ A_{z} \end{array}\right]}^{T}\cdot \left[\begin{array}{c} t_{y}\\ t_{z} \end{array}\right]\approx A_{x}\cdot t_{x}$$

The values among $A_{x},A_{y},A_{z}$ can be calculated, whereby if $A_{i}$ is the minimum value, the corresponding $t_{i}$ = 1 and the other two can be solved by matrix inversion. Then, the obtained t can be brought into Equation (4), and the value calculated by the coordinates of each matching point are regarded as the error. If the error satisfies certain conditions, the matching point is set as inliers. Finally, an inlier set will be obtained, and all inliers in the set will be substituted into Equation (5) to recalculate the new model by the least squares method. The model will be used to compute the final sum of error. Over multiple iterations, the inliers set with the smallest sum of errors is selected, thereby achieving the effect of removing the outliers. Additionally, performing circular matching [33] further removes the outlier matching step generated in the feature tracking and stereo matching between the previous and current stereo image pairs. Through the above steps, the mismatch of feature points is reduced, thereby improving the robustness and accuracy of the system.

### 3.2. IMU Measurement Model and Pre-Integration

**Figure 2:**
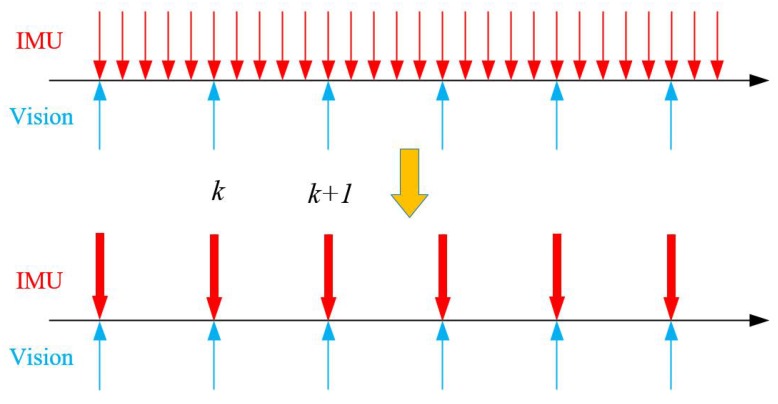
IMU pre-integration diagram.

As shown in Figure 2, the measurement rate of IMU is much faster than that of the visual camera. To simultaneously optimize the constraints of vision and IMU in a single framework, it is necessary to integrate the measurements of many IMUs between two adjacent visual keyframes into one constraint. Therefore, the theory of the pre-integration formula based on SO3 manifolds (18) was used in this paper.

The IMU measures acceleration and angular velocity in three directions within the inertial system by a three-axis accelerometer and a three-axis gyroscope. The IMU measurement is often affected by Gaussian white noise and zero offsets. The noise model of IMU can be expressed by the following formula:

(7) $${\tilde{\omega }}_{B}\left(t\right)=\omega_{B}\left(t\right)+b_{g}\left(t\right)+\eta_{g}\left(t\right)$$

(8) $${\tilde{a}}_{B}\left(t\right){=R}_{WB}^{T}\left(a_{W}\left(t\right)-g_{W}\right)+b_{a}\left(t\right)+\eta_{a}\left(t\right)$$

where ${\tilde{\omega }}_{B}\left(t\right)$ is the angular velocity measured by the gyroscope; ${\tilde{a}}_{B}\left(t\right)$ is the acceleration measured by the accelerometer; $\omega_{B}\left(t\right)$ and $a_{B}\left(t\right)$ are the real angular velocity and the real acceleration; b and η represent the corresponding zero-bias and white Gaussian noise, respectively.

To calculate the motion of the robot from the measured values of the IMU, the following kinematic models need to be introduced.

(9) $${\dot{R}}_{WB}=R_{WB}\omega_{B}^{\wedge }{\dot{v}}_{W}=a_{W}{\dot{p}}_{W}=v_{W}.$$

where ${\dot{R}}_{WB}$, ${\dot{v}}_{W}$ and ${\dot{p}}_{W}$ respectively represent the derivatives of the rotation matrix $R_{WB}$, the velocity vector $v_{W}$ and the translation vector $p_{W}$ with respect to time. Equation (9) describes the change in pose and velocity of the IMU in differential form. To obtain the value of the state of the IMU at each moment, the integration to Equation (9) needs to be determined. The pose and velocity of the IMU at time interval [t,t+Δt] can be described as follows:

(10) $$R_{WB}\left(t+\Delta t\right)=R_{WB}\left(t\right)Exp\left(\omega_{B}\left(t\right)\Delta t\right)\;v_{W}\left(t+\Delta t\right)=v_{W}\left(t\right)+a_{W}\left(t\right)\Delta t\;p_{W}\left(t+\Delta t\right)=p_{W}\left(t\right)+v_{W}\left(t\right)+\frac{1}{2}a_{W}\left(t\right)\Delta t^{2}$$

where Δt is the time interval between two adjacent IMU measurements. According to Equations (7) and (8), $\omega_{B}\left(t\right)$ and $a_{B}\left(t\right)$ in Equation (10) can be rewritten as the following forms.

(11) $$R\left(t+\Delta t\right)=R\left(t\right)Exp\left(\left(\tilde{\omega }\left(t\right)-b_{g}\left(t\right)-\eta_{g}\left(t\right)\right)\Delta t\right)\;v\left(t+\Delta t\right)=v\left(t\right)+g\Delta t+R\left(t\right)\left(\tilde{a}\left(t\right)-b_{a}\left(t\right)-\eta_{a}\left(t\right)\right)\Delta t\;p\left(t+\Delta t\right)=p\left(t\right)+v\left(t\right)\Delta t+\frac{1}{2}g\Delta t^{2}+\frac{1}{2}R\left(t\right)\left(\tilde{a}\left(t\right)-b_{a}\left(t\right)-\eta_{a}\left(t\right)\right)\Delta t^{2}$$

It is worth mentioning that the subscript of the reference coordinate system in Equation (11) is hidden for the legibility of the formula.

Based on Equation (11), the positional relationship of the inertial component at the adjacent Δt can be obtained. The sampling frequency of the IMU can be optimized if a state is added that needs to be evaluated every time interval in the collection of IMU data, which, however, will result in a slow calculation and arduous process of the IMU data. The IMU pre-integration method combines the IMU measurements between two adjacent visual keyframes i and j into a composite term that constitutes the motion constraints of two adjacent visual keyframes. Assuming that the IMU is synchronized with the visual frame detection time and the measurement data is acquired at discrete time *k*, the pose relationship between the two keyframes *k* = *i* and *k* = *j* can be obtained by the IMU measurement. To avoid the situation where the estimation of the initial frame of the pose estimation leads to the recalculation of the integral, this paper uses the incremental expression, which is defined as

(12) $$\Delta R_{ij}=R_{i}^{T}R_{j}=\prod_{k=i}^{j-1}Exp\left(\left({\tilde{\omega }}_{k}-b_{k}^{g}-\eta_{k}^{g}\right)\Delta t\right)\;\Delta v_{ij}=R_{i}^{T}\left(v_{j}-v_{i}-g\Delta t_{ij}\right)=\sum_{k=i}^{j-1}\Delta R_{ik}\left({\tilde{a}}_{k}-b_{k}^{a}-\eta_{k}^{a}\right)\Delta t\;\Delta p_{ij}=R_{i}^{T}\left(p_{j}-p_{i}-v_{i}\Delta t_{ij}-\frac{1}{2}g\Delta t_{ij}^{2}\right)=\sum_{k=i}^{j-1}\left[\Delta v_{ik}\Delta t+\frac{1}{2}\Delta R_{ik}\left({\tilde{a}}_{k}-b_{k}^{a}-\eta_{k}^{a}\right)\Delta t^{2}\right]$$

It should be noted that the IMU biases are regarded as being constants in the time interval Δt. However, the estimated biases are changed with a small amount of δb during optimization. The Jacobians $J_{\left(\cdot \right)}^{g}$ and $J_{\left(\cdot \right)}^{a}$ are employed to account for a first-order approximation of the effect of changing the biases without explicitly recomputing the pre-integration. The pose and velocity can be described as

(13) $$R_{WB}^{i+1}=R_{WB}^{i}\Delta R_{i,i+1}Exp\left(J_{\Delta R}^{g}b_{g}^{i}\right)\;v_{B}^{i+1}=v_{B}^{i}+g_{W}\Delta t_{i,i+1}+R_{WB}^{i}\left(\Delta v_{i,i+1}+J_{\Delta v}^{g}b_{g}^{i}+J_{\Delta v}^{a}b_{a}^{i}\right)\;R_{WB}^{i+1}=R_{WB}^{i}\Delta R_{i,i+1}Exp\left(J_{\Delta R}^{g}b_{g}^{i}\right)\;v_{B}^{i+1}=v_{B}^{i}+g_{W}\Delta t_{i,i+1}+R_{WB}^{i}\left(\Delta v_{i,i+1}+J_{\Delta v}^{g}b_{g}^{i}+J_{\Delta v}^{a}b_{a}^{i}\right)$$

## 4. Visual-Inertial Initialization

The primary purpose of Visual-Inertial SLAM system initialization is to obtain the parameters necessary for the system to optimize and the initial values of the state. Since the stereo inertial tightly coupled system is a highly nonlinear system, it becomes very sensitive to specific initial values. The initialization quality directly affects the robustness and accuracy of the localization of the entire tightly coupled system. Therefore, it is necessary to initialize the system properly to provide correct parameters and initial values.

In the process of initialization, the information that needs to be initialized or estimated can be divided into two categories. One is the parameters that almost remain unchanged during the system operation, such as absolute scale and gravity acceleration. Another is the initial values of the system’s starting state quantities, including the pose and velocity information of the first few frames, the position of the 3D landmark points, and the zero offset of the IMU accelerometer and gyroscope. The initialization can be separated into two processes. Firstly, the stereo visual initialization can initialize the initial frame pose and the three-dimensional landmark position information based on the sliding window. Then, the visual inertia joint initialization can initialize the absolute scale, gravity acceleration, and camera speed information.

### 4.1. Stereo Vision Initialization Based on a Sliding Window

In the process of stereo visual initialization based on a sliding window, an up-to-scale purely visual structure of the sliding window is constructed to restore the information of the initial frames pose and the three-dimensional landmark point position.

Two frames are first selected that have sufficient feature disparity in the sliding window. Next, the eight-point method of the polar geometry recovery pose is used to recover the essential matrix E. The scale of the translation transformation is fixed, and E is used to retrieve the motion pose and triangulate the 3D map points. After initializing a batch of 3D points, the perspective-n-point (PnP) method is employed to solve the pose information of the remaining frames in the sliding window. A global full bundle adjustment is used to minimize the total reprojection error for all feature observations in all frames. At this point, the measurement can obtain the pose information and the three-dimensional information of the feature points. Since the external parameters $T_{CB}=\left(R_{CB},p_{CB}\right)$ between the camera and the IMU are known, all variables can be converted to representations in the IMU coordinate system:

(14) $$R_{WB_{k}}^{}=R_{CC_{k}}R_{CB}^{-1}$$

(15) $$sp_{B_{k}}^{W}=sp_{C_{k}}^{W}+R_{WC}p_{B}^{C}$$

where *s* is an unknown scale factor that aligns the visual structure to the metric scale, and feature positions ${\left(\cdot \right)}^{W}$ are represented with respect to the world coordinate system.

### 4.2. Visual Inertial Joint Initialization

Through the visual inertia joint initialization, the absolute scale, gravity acceleration, camera state speed information, and IMU zero offset can be initialized.

#### 4.2.1. Gyroscope Bias Estimation

The IMU bias is firstly initialized under the assumption that the IMU gyroscope zero offset $b_{g}$ is constant in the current window. Considering the adjacent kth and k+1th frames in the window, in the stereo visual initialization of the previous step, we can obtain the rotations $R_{k}$ and $R_{k+1}$ in their relative world coordinate system. The angular velocity among the results of IMU pre-integration can be derived from Equation (12). The gyroscope bias can be predicted by minimizing the error of the term.

(16) $$\underset{b_{g}}{\min}{\sum_{k}^{N-1}\left\|Log\left({\left(\Delta R_{k,k+1}Exp\left(J_{\Delta R}^{g}b_{g}\right)\right)}^{T}R_{BW}^{k+1}R_{WB}^{k}\right)\right\|}^{2}$$

By solving this least squares problem, we can estimate the gyroscope zero offsets $b_{g}$.

#### 4.2.2. Accelerometer Bias, Gravity and Metric Scale Estimation

It is difficult to solve the accelerometer bias during the initialization process because of g and the accelerometer bias are measured simultaneously. However, the accelerometer bias $b_{a}$ has little effect on system stability, a rough initial guess can thus be made, and the bias will be continuously optimized during subsequent optimizations. Therefore, in the initialization step, we set the accelerometer offset $b_{a}$ to zero.

The remaining estimated parameters: the speed of the state, the gravity, and the scale factor are defined as the variables:

(17) $$\chi_{v,s}=\left[v_{B_{0}},v_{B_{1}},\cdots v_{B_{n}},g,s\right]$$

where $v_{B_{k}}$ is velocity in the body frame while tracking the *k*th image, *g* is the gravity vector in the world frame, and *s* is the scale of Visual-Inertial SLAM system to metric units.

Considering two consecutive frames $b_{k}$ and $b_{k+1}$, we can develop the following linear measurement model from Equations (12), (14) and (15):

(18) $$\begin{array}{cc} {\hat{z}}_{b_{k,k+1}} & =\left[\Delta p_{k,k+1}-R_{WB}^{k}{}^{T}\left(R_{WC}^{k+1}-R_{WC}^{k}\right)p_{B}^{C}\right]\\ & =\left[-R_{WB}^{k}\Delta t_{k,k+1}\;\;R_{WB}^{k}{}^{T}\left(p_{C_{k+1}}^{W}-p_{C_{k}}^{W}\right)\;\;-\frac{1}{2}R_{WB}^{k}{}^{T}\Delta t_{k,k+1}\right]\chi_{v,s}\\ & =H_{k,k+1}\chi_{v,s} \end{array}$$

The initial value can be guessed by solving the following least squares problem:

(19) $$\underset{v,s,g}{\min}{\left\|{\hat{z}}_{b_{k,k+1}}\;\;\;-H_{k,k+1}\chi_{v,s}\right\|}^{2}$$

The speed of each local frame, the gravity vector (including direction and intensity), and the scale factor can be obtained.

## 5. Tightly Coupled Stereo Visual-inertial Odometry

After the state initialization, a highly accurate state is estimated using a sliding window estimator. A state estimator based on the combination of stereo vision and IMU is described in this section. The state estimator can be regarded as a backend based on nonlinear graph optimization. It optimizes the coordinates of the 3D landmark points and the pose, velocity and IMU bias of the inertial camera. In this section, the state vector of the system estimation is defined firstly, then the expression of the visual inertia optimization term is given. After that, the explicit calculation formulas of the visual error term and the inertia error term are provided, and finally, the system’s marginalization method is presented.

### 5.1. System State Vector

First, the state vectors of the system are defined. The state vector to be estimated are the state variable χ of the inertial camera at the image time *k*, including position $p_{B}$, pose $R_{WB}$, velocity $v_{B}$, gyroscope bias $b_{g}$, and accelerometer bias $b_{a}$.

(20) $$\chi =\left[x_{0},x_{1}\cdots x_{n}\right]$$

(21) $$x_{n}=\left[R_{WB},p_{B},v_{B},b_{g},b_{a}\right]$$

### 5.2. IMU and Visual Error Term

In the traditional visual SLAM or visual odometers, nonlinear optimization is used to obtain the best estimate of camera pose and 3D landmark points by minimizing the reprojection error of feature points in the camera frame. Once inertial measurements are introduced, constraints are applied to the camera’s continuous motion poses, the IMU speed and the estimated IMU bias for continuous time, resulting in an increase of the system state variables. The IMU error term can be defined as:

(22) $$e_{IMU}({\hat{z}}_{b_{k,k+1}},\chi )=\rho \left(\left[e_{R}^{T}e_{V}^{T}e_{p}^{T}\right]\sum_{I}{\left[e_{R}^{T}e_{V}^{T}e_{p}^{T}\right]}^{T}\right)+\rho \left(e_{b}^{T}\sum_{R}e_{b}\right)\;e_{R}=Log\left({\left(\Delta R_{k,k+1}Exp\left(J_{\Delta R}^{g}b_{g}^{k+1}\right)\right)}^{T}R_{BW}^{k+1}R_{WB}^{k}\right)\;e_{v}=R_{BW}^{k}\left(v_{B}^{k+1}-v_{B}^{k}-g\Delta t_{k,k+1}\right)-\left(\Delta v_{k,k+1}+J_{\Delta v}^{g}b_{g}^{k+1}+J_{\Delta v}^{a}b_{a}^{k+1}\right)\;e_{v}=R_{BW}^{k}\left(v_{B}^{k+1}-v_{B}^{k}-g\Delta t_{k,k+1}\right)-\left(\Delta v_{k,k+1}+J_{\Delta v}^{g}b_{g}^{k+1}+J_{\Delta v}^{a}b_{a}^{k+1}\right)\;e_{p}=R_{BW}^{k}\left(p_{B}^{k+1}-p_{B}^{k}-v_{B}^{k}\Delta t_{k,k+1}-\frac{1}{2}g\Delta t_{k,k+1}^{2}\right)-\left(\Delta p_{k,k+1}+J_{\Delta p}^{g}b_{g}^{k+1}+J_{\Delta p}^{a}b_{a}^{k+1}\right)\;e_{b}=b^{k+1}-b^{k}$$

where ρ is the Huber robust cost function, and $\sum_{I}$ is the information matrix of the pre-integration and $\sum_{R}$ is the bias random walk.

Consider the feature in the $j^{th}$ image, and the reprojection error of feature points $e_{proj}$ for a given match k to be defined as follows:

(23) $$e_{proj}\left(k,j\right)=\rho \left({\left(x^{k}-\pi \left(X_{c}^{k}\right)\right)}^{T}\sum_{k}\left(x^{k}-\pi \left(X_{c}^{k}\right)\right)\right)\;X_{C}^{k}=R_{CB}R_{BW}^{j}\left(X_{W}^{k}-p_{WB}^{j}\right)+p_{CB}$$

where $x^{k}$ is the location of the observation keypoint in the image, $X_{c}^{k}$ is the map point in world coordinates, and $p_{WB}^{j}$ represents the translation of IMU relative to the world coordinate system at $j^{th}$ frames, and $\sum_{k}$ is the information matrix associated with the keypoint scale.

### 5.3. Marginalization

As time goes by, the feature points and camera poses will accumulate, and the amount of optimization calculation will increase accordingly. Because the dimension of the visual inertia system to be optimized is more than visual system, the number of variables to be optimized is large. If the estimated variables are not limited, the calculation load will surge with time. Therefore, the non-linearization is performed while the state of the estimated system is marginalized, i.e., the time-limited keyframes are removed without changing the consistency of the estimation.

The visual-inertial optimization term is a least squares problem, which can generally be solved by the Gauss-Newton iteration method. This can be defined as:

(24) Hδx=b

Suppose $\delta x_{a}$ is the state variable to be marginalized, and $\delta x_{b}$ is the state variable to be retained. Depending on the conditional independence, we can simplify the process of marginalization as follows:

(25) $$\left[\begin{array}{cc} H_{a} & H_{b}\\ H_{b}^{T} & H_{d} \end{array}\right]\left[\begin{array}{c} \delta x_{a}\\ \delta x_{b} \end{array}\right]=\left[\begin{array}{c} b_{a}\\ b_{b} \end{array}\right]$$

$\delta x_{a}$ in the above formula is the variable for marginalization, such as a camera pose. We cannot directly delete $\delta x_{b}$ and its related landmark points, because this would reduce the constraint information. Therefore, the following Schur complement can be used to eliminate the element:

(26) $$\left(H_{c}-H_{c}H_{a}^{-1}H_{b}\right)\delta x_{b}=b_{b}-H_{c}H_{a}^{-1}b_{a}$$

where $H_{c}-H_{c}H_{a}^{-1}H_{b}$ and $b_{b}-H_{c}H_{a}^{-1}b_{a}$ are marginalized *H* matrices and error quantities, and state variables $\delta x_{a}$ are marginalized. By repeating the marginalization, as the new state variable is added, the number of state variables to be optimized remains constant, which dramatically decreases the amount of computation.

## 6. Experimental Results

**Figure 3:**
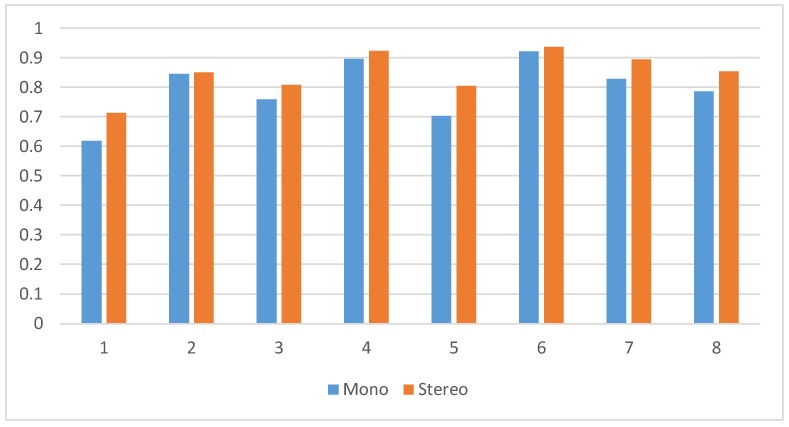
Mismatch accuracy of different types of cameras.

To validate the effectiveness of the proposed reduced mismatch algorithm, the EuRoC dataset of images was used to test the visual front end. Two consecutive frames of images were randomly selected, a total of eight groups (among monocular image using the left camera image), keypoints were extracted for both the monocular image and stereo image, and the RANSAC algorithm was then used to reduce the mismatch. By calculating the comparison between the number of points removed and the original untreated points, it was found that the image matching accuracy after stereo processing was high, with the specific results shown in Figure 3.

**Figure 4:**
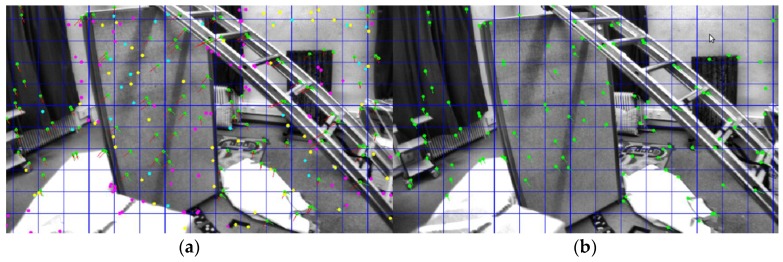
The tracking of the visual front end: (**a**) left image; (**b**) right image.

An experiment was performed to validate the proposed method. A comparison with the existing state-of-art visual-inertial odometry algorithms VINS-Mono and S-MSCKF through the EuRoC MAV Visual-Inertial datasets was also made. The EuRoC datasets were retrieved by a microdrone. The UAV was equipped with sensors such as stereo cameras (Aptina MT9V034, 20FPS) and IMU (ADIS16448, 200 Hz), and the real-time ground truth states were acquired by the motion capture device. The movement of the drone had a large rotation and a significant change in light intensity, which was challenging for our vision-based odometry. The tracking of the visual front end is shown in Figure 4.

**Figure 5:**
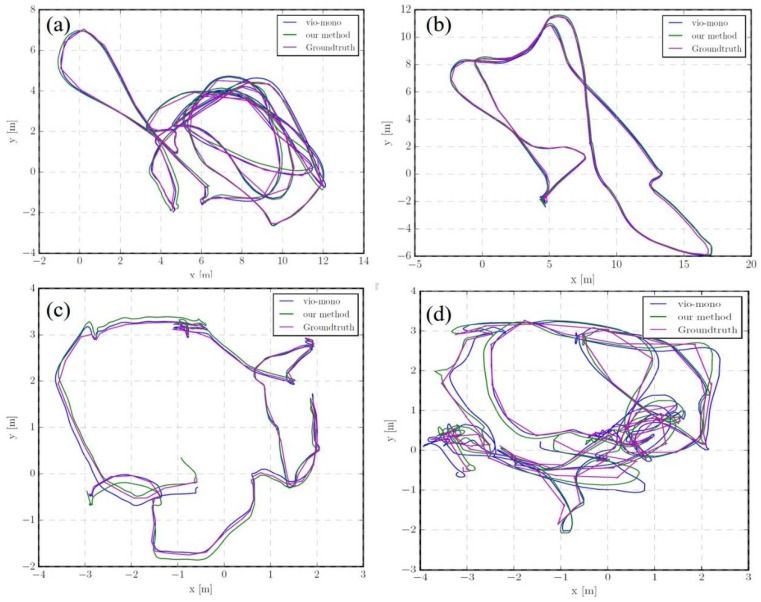
Trajectory compared with VINS in (**a**) MH_03_median, (**b**) MH 05 difficult, (**c**) V2_01_easy, and (**d**) V2_02_median.

In these experiments, the proposed method was compared with VINS-Mono and S-MSCKF, which were tested on a desktop computer. The desktop CPU was equipped with quad-core i7-6700 3.4 Hz, the memory is 8 GB. The EuRoC datasets have eleven datasets, and four representative sequences, including MH_03_median, MH 05 difficult, V2_01_easy, and V2_02_median, were used in the dataset, to compare with VINS-Mono. Figure 5 shows the trajectory for the sequence MH_03_median, MH 05 difficult, V2_01_easy, and V2_02_median. Obviously, the estimated trajectory by our method is even closer to the real one.

**Figure 6:**
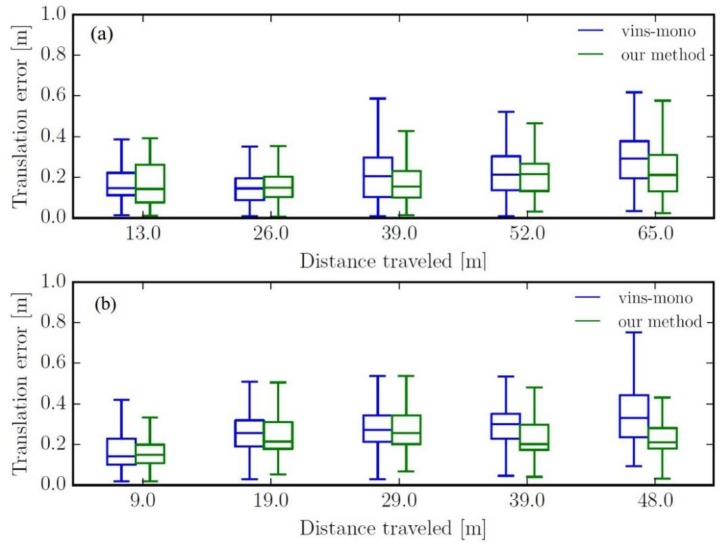
Translation error plot: (**a**) MH_03_median, (**b**) MH_05_median.

The relationship between the translation error and the distance is shown in Figure 6. Due to space limitations, only the results of MH_03_median and MH_05_difficult are demonstrated. In the error graph, the proposed method produces the smallest translation error as the distance increases. Overall, the estimated error of this method is less than 5% on all sequences.

The relationship between the translation error and the distance is shown in Figure 6. Due to space limitations, only the results of MH_03_median and MH_05_difficult are demonstrated. In the error graph, the proposed method produces the smallest translation error as the distance increases. Overall, the estimated error of this method is less than 5% on all sequences.

In addition, we also compared our method with VINS-Mono in terms of CPU resource requirements, and the results are listed in Table 1. As can be seen, our method is comparable to the VINS-Mono on the CPU load, but the memory utilization statistics appears relatively better. Thus, from Figure 5 and Figure 6 and Table 1, it can be concluded that our method obtains comparable accuracy at the start of the trajectory, but in a more efficient manner, and as the distance increases, the method begins to have much better performance than VINS-Mono.

To evaluate the performance of this method, the tool evo (https://github.com/MichaelGrupp/evo) is used to determine the Root Mean Square Error (RMSE) of the experimental results, and the results are shown in Table 2. Notably, stereo feature matching failed because of the continuous inconsistency in brightness between stereo images in V2_03_difficult. Therefore, V2_03_difficult sequence was not included here. In the initial stage, the accuracy of our method is not greatly superior to VINS-Mono. As the flight distance increases, however, the error of our method becomes significantly lower than that of VINS, ensuring comparably accurate results over long paths. In the end, our method maintains the best performance, with an average RMSE of 0.075 m, as demonstrated in Table 2. It should be emphasized here that our method successfully recovers the metric scale, which, however, could not be achieved just by using the monocular camera. Compared to the optimization-based monocular SLAM, our method is more accurate than VINS in most scenarios except for individual scenes. For filter-based S-MSCKF, its accuracy is much lower than our method and VINS, which proves that optimization-based methods have greater potential than filter-based methods. It is worth mentioning that VINS-Mono, slightly inferior to our proposed algorithm, has outperformed both OKVIS and MSCKF in our results as shown in Table 2. These findings may not agree with the published results in [16] as VINS-Mono in their tests are running at a relatively low frequency without loop closure detection. When playing at full strength, VINS-Mono has experimentally been proved to be state-of-the-art and perform better than the widely used VIO systems, including OKVIS and MSCKF [33]. In summary, the optimization-based method has high localization accuracy and low memory utilization, while the filter-based method has advantages in computing resources. This is a remarkable result of our optimization-based stereo method, compared to the filter-based stereo VIO (16) and the optimization-based monocular VIO (19).

## 7. Conclusions

In this paper, a robust stereo visual-inertial tightly coupled localization method was proposed. The method outputs the pose information of the drone in real-time through the onboard sensor, namely, the camera and the inertial sensing unit. The accuracy, real-time and robustness of the proposed method are validated by comparisons with the state of art open source algorithms in real-time operation in the EuRoC dataset. In most cases, this method shows better performance and lower computational cost, when running on low-computing devices. In future work, experiments will be conducted based on the drones, in combination with the proposed localization method as well as obstacle avoidance algorithms to achieve autonomous navigation.

## Figures and Tables

**Table 1:** CPU load and memory utilization statistics of the algorithms (%).

Sequence	CPU Load		Memory Utilization	
	Ours	VINS-Mono	Ours	VINS-Mono
V1_01_easy	**40.25**	43.14	**26.18**	34.09
V1_02_medium	**34.05**	36.78	**25.98**	33.22
V1_03_difficult	36.72	**34.83**	**26.72**	34.75
V2_01_easy	42.32	**39.45**	**26.04**	32.02
V2_02_medium	45.88	**42.37**	**25.65**	34.48
V2_03_difficult	/	40.76	**/**	34.18
MH_01_easy	40.59	**38.87**	**26.24**	34.08
MH_02_easy	**37.06**	39.08	**26.43**	34.11
MH_03_medium	**38.57**	40.69	**26.06**	35.04
MH_04_difficult	44.02	**41.33**	**26.28**	34.77
MH_05_difficult	45.38	**40.9**	**26.2**	37.8
Average	40.48	**39.84**	**26.18**	34.41

The numbers in bold indicate the lower computational cost of the algorithms.

**Table 2:** RMSE of the algorithms (m).

Sequence	Ours	VINS-Mono	S-MSCKF	OKVIS
V1_01_easy	**0.039**	0.043	0.065	0.208
V1_02_medium	0.051	**0.047**	0.154	0.19
V1_03_difficult	0.105	**0.09**	0.281	0.195
V2_01_easy	**0.051**	0.062	0.069	0.172
V2_02_medium	**0.11**	0.121	0.147	0.181
V2_03_difficult	/	**0.113**	/	0.327
MH_01_easy	**0.053**	0.066	0.215	0.169
MH_02_easy	**0.078**	0.081	0.226	0.193
MH_03_medium	**0.041**	0.045	0.202	0.285
MH_04_difficult	0.134	**0.129**	0.323	0.386
MH_05_difficult	**0.089**	0.095	0.224	0.455
Average	**0.075**	0.081	0.19	0.251

The numbers in bold indicate the smallest values of RMSE of the algorithms.
